# A High-Throughput Screening Platform of Microbial Natural Products for the Discovery of Molecules with Antibiofilm Properties against *Salmonella*

**DOI:** 10.3389/fmicb.2017.00326

**Published:** 2017-03-02

**Authors:** Sonia Paytubi, Mercedes de La Cruz, Jose R. Tormo, Jesús Martín, Ignacio González, Victor González-Menendez, Olga Genilloud, Fernando Reyes, Francisca Vicente, Cristina Madrid, Carlos Balsalobre

**Affiliations:** ^1^Department de Genètica, Microbiologia i Estadística, Facultat de Biologia, Universitat de BarcelonaBarcelona, Spain; ^2^Fundación MEDINA, Parque Tecnológico de Ciencias de la SaludGranada, Spain

**Keywords:** biofilm, *Salmonella*, HTS, natural products, patulin, antimicrobial

## Abstract

In this report, we describe a High-Throughput Screening (HTS) to identify compounds that inhibit biofilm formation or cause the disintegration of an already formed biofilm using the *Salmonella* Enteritidis 3934 strain. Initially, we developed a new methodology for growing *Salmonella* biofilms suitable for HTS platforms. The biomass associated with biofilm at the solid-liquid interface was quantified by staining both with resazurin and crystal violet, to detect living cells and total biofilm mass, respectively. For a pilot project, a subset of 1120 extracts from the Fundación MEDINA's collection was examined to identify molecules with antibiofilm activity. This is the first validated HTS assay of microbial natural product extracts which allows for the detection of four types of activities which are not mutually exclusive: inhibition of biofilm formation, detachment of the preformed biofilm and antimicrobial activity against planktonic cells or biofilm embedded cells. Currently, several extracts have been selected for further fractionation and purification of the active compounds. In one of the natural extracts patulin has been identified as a potent molecule with antimicrobial activity against both, planktonic cells and cells within the biofilm. These findings provide a proof of concept that the developed HTS can lead to the discovery of new natural compounds with antibiofilm activity against *Salmonella* and its possible use as an alternative to antimicrobial therapies and traditional disinfectants.

## Introduction

Biofilms are defined as complex microbial communities embedded in a self-produced extracellular polymeric matrix that attach to surfaces and are the predominant mode of microbial growth in nature (Steenackers et al., 2012). Biofilm formation is a developmental process which starts with a motile cell approaching and adhering reversibly to a surface (O'Toole et al., 2000). In a next step, irreversible attachment occurs with the subsequent development of microcolonies which produce an extracellular matrix, promoting the development of the mature three-dimensional biofilm (O'Toole et al., 2000). During biofilm dispersal, cells undergo controlled lysis and escape from the microbial community (O'Toole et al., 2000). Biofilms become a threatening concern since they might provide a reservoir of pathogenic bacteria, increasing the risk of microbial contamination, and leading to critical problems in terms of public health and economical lost (Shi and Zhu, 2009). They are recalcitrant, highly resistant to antimicrobials and disinfectants and consequently are extremely difficult to eradicate (O'Toole et al., 2000; Bridier et al., 2011). There are two main mechanisms that contribute to such recalcitrance, (i) the biofilm matrix might prevent the diffusion of the biocides toward the cells and (ii) some cells within a biofilm show very low metabolic activity (dormant cells) becoming resistant to the action on many biocides (Otto, 2008; Lister and Horswill, 2014). It has been estimated that the minimum concentration of biocide required to kill bacteria within a biofilm may be up to 100–1000 times higher compared to planktonic cells (Costerton et al., 1995; Stoodley et al., 2002; Høiby et al., 2010). Since most antimicrobials currently in use show a poor activity against bacteria embedded in biofilms (Høiby et al., 2010), search for new molecules with antibiofilm properties is a thrilling challenge.

During food processing, contact of food products with biofilm contaminated surfaces is a relevant mode of transmission of pathogens and spoiling bacteria (Chmielewski and Frank, 2003). Adhesion of *Salmonella* to food surfaces was the first published report on foodborne bacterial biofilm (Duguid et al., 1966). *Salmonella enterica* serovar Enteritidis is one of the causal agents of salmonellosis, a food-borne disease with high incidence worldwide, affecting tens of millions of human cases, according to WHO (Kirk et al., 2015). Every year, *Salmonella* spp. non-typhoidal is estimated to cause up to 1,200,000 cases in USA, with 23,000 hospitalizations and 450 deaths (Scallan et al., 2011). The high persistence of non-typhoidal *Salmonella* relies on its ability to form biofilms (Steenackers et al., 2012).

Within this context, natural products (NPs) yielded by bacterial, fungal, plant, and marine sources, represent a large family of diverse chemical entities with a wide variety of biological activities that have found multiple uses, remarkably in human and veterinary medicine and in agriculture (Katz and Baltz, 2016). They serve as a valuable source for novel molecular scaffolds in drug development, and around 65% of all approved drugs are classified as NPs or are inspired by a NP core (Newman and Cragg, 2012). As compared to synthetic molecules, NPs possess structural diversity and complexity, more stereogenic centers and fewer halogen or nitrogen atoms (Chen et al., 2015). These properties are expected to contribute to the ability of NPs collections to provide hits even against the more difficult screening targets (Harvey et al., 2015). In this report, we describe the development of a robust and validated experimental approach using a High-Throughput Screening (HTS) platform to identify natural compounds that either can inhibit the formation or can eliminate or disperse *S. enterica* preformed biofilms. *S. enterica* serovar Enteritidis strain 3934, a clinical isolate capable of forming biofilms at the air-liquid interface (pellicle) in rich salt-free media, was selected as a model organism (Solano et al., 1998). Although relevant pathogens form pellicle biofilm, there are not reliable methods available to accurately quantify pellicle biofilms formed in small cultures. Most of the studies are based in visual inspection of the pellicle and qualitative comparison among samples. For the purpose of evaluating the effect of a vast number of compounds on *Salmonella* biofilms, a robust and reliable methodology to quantify biofilms was needed. To do that, the growth conditions in which *S. enterica* serovar Enteritidis forms a solid-liquid interface biofilm suitable for an easy and reliable quantification during the HTS were validated. This approach has led us to the identification of compounds active against biofilm formation and compounds that promote dispersion of already existing biofilms. The experimental layout has been successfully applied to screen 1120 natural extracts from actinomycetes and fungi of the Fundación MEDINA's collection. Five of these extracts have been selected attending to its antibiofilm activity. Amongst them, using a bioassay-guided fractionation approach, we have identified patulin, a mycotoxin that shows a great potential as antimicrobial agent against biofilm embedded bacterial cells.

## Materials and methods

### Bacterial strains and culture media

*S. enterica* serovar Enteritidis strain 3934 (Solano et al., 1998), a clinical isolate described previously, was used for HTS experiments. The clinical isolates of *S. enterica* serovar Enteritidis HC164372.7 and *S. enterica* serovar Typhimurium HC142156206 (Microbiology Laboratory at the Hospital Clinic of Barcelona, Spain) and strain *S. enterica* serovar Typhimurium ATCC 14028 (obtained from the American Type Culture Collection) were used to validate the effect of culture media on *Salmonella* biofilms.

Cultures of the different *S. enterica* strains were grown in LB-agar (10 g/l NaCl, 10 g/l tryptone, 5 g/l yeast extract, and 15 g/l agar), CFA (10 g/l casamino acids, 1.5 g/l yeast extract, 0.4 mM MgSO_4_, and 0.4 mM MgCl_2_; Suzuki et al., 2002) or E medium. E minimal medium was prepared by diluting to 1X a stock of Vogel-Bonner salts 50X (0.8 mM MgSO_4_, 9.5 mM citrate, 57 mM K_2_HPO_4_, and 17 mM NaNH_4_HPO_4_; Vogel and Bonner, 1956) with sterile Milli-Q water (Milli-Q Reference, Millipore).

### HTS assay conditions

HTS was performed in sterile, flat-bottomed, 96-well-polystyrene micro-well-plates (Nunclon Δ surface; Nunc, Roskilde, Denmark). For biofilm formation assays, each 96-well-plate was loaded as follows: 10 μl of antibiotics, dimethyl sulfoxide (DMSO), or extracts (2X whole broth equivalents, WBE) from the Fundación MEDINA's collection (Genilloud et al., 2011; Monciardini et al., 2014; http://www.medinadiscovery.com) was added per well using an automated Aquarius Multi-channel Pipettor (Tecan) and a volume of 90 μl of the E minimal medium containing the *Salmonella* inoculum was added using Multidrop Combi (Thermo). The optimum inoculum per well was ~1.4 × 10^6^ cfu/well, which corresponds to an OD_612_ of 0.02. Plates were incubated for 72 h at 25°C in static conditions inside a plastic bag with wet cellulose paper to maintain the proper humidity levels. For the “biofilm formation assay,” total biomass (planktonic and biofilm) was monitored by determination of the OD_612_ before and after incubation using an EnVision Multilabel Plate Reader (PerkinElmer). Next, staining with resazurin (RSZ) and crystal violet (CV) was performed. For “biofilm dispersal assay,” *Salmonella* cells were inoculated on 96-well-microplates as mentioned above, with the difference that no extract was added in order to obtain a mature biofilm. After incubation (72 h at 25°C), plates were washed twice with sterile Milli-Q water, pat dried and natural extracts, antibiotics and DMSO were loaded as stated above for the biofilm formation assay. Plates were incubated for an additional 24 h and total biomass was monitored by determination of the OD_612_, followed by the determination of the viability by RSZ staining and of biofilm biomass by CV staining.

Each 96-well-plate contained a set of control wells located in the left and right columns (Figure S1). The control wells were established as follows: (1) four wells without testing sample (inoculum + 2% DMSO, as natural extracts contain a 20% DMSO) were the untreated controls; (2) four wells without inoculum (medium + 2% DMSO) were the negative controls; and (3) eight wells correspond to the antibiotic kanamycin dose-response curve (62–0.5 μg/ml, 1:2 serial dilutions).

### RSZ metabolic assay

For quantitative measurement of the living cells of the biofilm, RSZ staining, that monitors the metabolic activity, was performed (O'Brien et al., 2000). Resazurin sodium salt (Sigma) was prepared in Milli-Q water at a concentration of 0.02% (w/v) and sterilized by filtration.

RSZ staining protocol was performed as described by Monteiro et al. (2012) with minor changes. First, plates were rinsed twice with distilled water and pat dried with paper towels. Next, 100 μl of E minimal medium and 25 μl of RSZ 0.02% was dispensed per well, incubated in the darkness for 1–2 h at 37°C in static conditions and fluorescence (excitation 570 nm, emission 615 nm) was monitored using an EnVision Multilabel Plate Reader.

### CV staining

For quantitative measurement of the biofilm biomass, CV staining was performed. CV binds to negatively charged molecules and can be used to stain and quantify total biomass comprising bacteria and extracellular polymeric substances (EPS) within a biofilm (Stepanovic et al., 2000). CV staining protocol was performed as described by Paytubi et al. (2014) with minor modifications. Briefly, plates were rinsed twice with distilled water and biofilms were fixed by heating at 80°C for 30 min. Next, 200 μl of a CV solution (1% w/v, prepared in Milli-Q water) was added to each well and incubated for 15 min, rinsed with water and air-dried. Hundred microliter of acetic acid 30% (v/v) was added to each well and shaken gently to ensure the complete solubilization of the CV. The OD_570nm_ of the resulting solution was determined.

### Fundación MEDINA's collection

For the primary screening campaign, a subset of 1120 microbial extracts from different modules of the MEDINA NPs collection was used. The microbial extracts were obtained from bacterial and fungal strains (560 of each) cultivated in different nutritional conditions and extracted with acetone (1:1) for 1 h in an orbital shaker. Extracts were then centrifuged at 1500 × g for 15 min and the supernatant concentrated to half of the original volume (2X WBE) in the presence of a final concentration of 20% DMSO. Extracts were stored at −20°C in 96-well ABgene v-bottom plates until needed.

### Data analysis

Extract activities were calculated automatically using the Genedata Screener software (Genedata AG, Basel, Switzerland), and the percentage inhibition of each sample (extract or compound) was determined by the following equations, depending on the type of staining protocol.

For RSZ staining, the percentage of inhibition was expressed as a function of the percentage of fluorescence emitted by resorufin (reduced RSZ that emits fluorescence) and was calculated as follows,

(1) $$\begin{array}{l} \%\text{ inhibition}=\left[100\times \left(\frac{\text{fluorescence intensity of }(\text{sample}-\text{negative control})}{\text{fluorescence intensity of }(\text{untreated control}-\text{negative control})}\right)\right]-100 \end{array}$$

The color of the wells was also visually recorded. A blue color reflected the absence of metabolic activity (no biofilm or dead cells in the biofilm). A fluorescent pink color (due to the presence of resorufin, obtained by reduction of RSZ) reflected metabolic activity (cells alive in the biofilm).

For CV staining, the percentage of inhibition was expressed as a function of the percentage of OD_570_ and was calculated as follows,

(2) $$\begin{array}{l} \%\text{ inhibition}=\left[100\times \left(\frac{{\text{OD}}_{570}\;\text{of }(\text{sample}-\text{negative control})}{{\text{OD}}_{570}\;\text{of }(\text{untreated control}-\text{negative control})}\right)\right]-100 \end{array}$$

For total biomass, the percentage of inhibition was expressed as a function of the percentage of OD_612_ and was calculated as follows,

(3) $$\begin{array}{l} \%\text{ inhibition}=100\times \left[1-\left(\frac{{\text{OD}}_{612}\;\text{of }[(\text{sample Tf}-\text{sample T}0)-(\text{negative control Tf}-\text{negative control T}0)]}{{\text{OD}}_{612}\;\text{of }[(\text{untreated control Tf}-\text{untreated control T}0)-(\text{negative control Tf}-\text{negative control T}0)]}\right)\right] \end{array}$$

Where Tf is the measurement at final time and T0 is measurement at time zero.

Using the data obtained with RSZ staining, extracts were selected when they displayed at least a 90% inhibition on biofilm formation assays. On the other hand, when looking at the effect of the extracts on preformed biofilm by the biofilm dispersal assay, a threshold of 45 and 35% inhibition was used for actinomycetes and fungal extracts, respectively.

The Genedata Screener software was used to calculate the RZ′ and Z′ factors, which predicts the robustness of an assay (Zhang et al., 1999).

### LC-MS and database matching of known secondary metabolites

LC-MS analyses in the low (LR) or high resolution (HR) mode were performed as previously described (Martín et al., 2014; Pérez-Victoria et al., 2016). Database searching was performed against the MEDINA proprietary database of microbial metabolites or the Chapman & Hall Dictionary of Natural Products (v25.1).

### Bioassay-guided extract fractionation

Culture broths generated were extracted by addition of a 1:1 volume of acetone. After agitation for 2 h and centrifugation, the acetone was removed under a nitrogen stream and the remaining solution was loaded onto an SP207ss cartridge (1.5 × 5 cm) that was washed with water (100 ml) and eluted with methanol (40 ml) and acetone (60 ml). The combined organic eluates were evaporated, the residue was dissolved in DMSO (400 μl), and 200 μl of this volume were subjected to semipreparative HPLC on a Gilson GX-281 apparatus equipped with a Zorbax SB C8 colum (9.8 × 250 mm) at a flow rate of 3.6 ml/min, using a gradient of H_2_O:CH_3_CN (5% CH_3_CN for 1 min, then to 100% CH_3_CN in 35 min, held at 100% CH_3_CN for 7 min and back to 5% CH_3_CN in 1 min) and UV detection at 210 and 280 nm was monitored. Eighty fractions were collected every 30 s from min 2 to 42. These fractions were evaporated, dissolved in 20% DMSO and subjected to bioactivity test.

### IC_50_, MIC, and MBEC determination

IC_50_ (Motulsky and Christopoulos, 2003), MIC (Minimal Inhibitory Concentration) (Clinical Laboratory Standards Institute, 2012), and MBEC (Minimal Biofilm Eradication Concentration) (Ceri et al., 1999) values were obtained from dose-response curves as follows. The initial concentrations of the different compounds and extracts were 1.66 mg/ml for patulin, 50 mg/ml for kanamycin, 10 mg/ml for chloramphenicol, 25 mg/ml for ciprofloxacin, and 2X WBE for extracts and the subsequent points were obtained by 2-fold serial dilution. Serial dilutions of the extracts were performed using 20% DMSO, to make a 2% final concentration of DMSO in the assay. Patulin (Fisher Scientific), kanamycin (Sigma-Aldrich), chloramphenicol (Sigma-Aldrich), and ciprofloxacin (Sigma-Aldrich) were disolved in ethyl acetate, sterile Milli-Q water, ethanol, and HCl 0.1N, respectively, and serial dilutions were done with the corresponding solvent. Once the dilutions were performed, 10 μl of each dilution was used for biofilm assays, following the same procedure as for the HTS assay. All experiments were performed in duplicate.

IC_50_ on both, biofilm formation assay and biofilm dispersal assay, was calculated using Graphpad Prism v5.01 software. MIC was determined by readings of OD_612_, CV, and RSZ. MBEC was determined by RSZ staining.

### Cytotoxicity assay

The cytotoxic activity of the different compounds was tested against the hepatic cell line HepG2 (Knowles et al., 1980) in the MTT colorimetric assay. MTT [3-(4,5-Dimethylthiazol-2-yl)-2,5-diphenyltetrazolium bromide] is a colorimetric assay for measuring the activity of cellular enzymes that reduce the tetrazolium dye, MTT, to its insoluble formazan, giving a purple color (Patel and Patel, 2011). This assay measure mitochondrial metabolic activity via NAD(P)H-dependent cellular oxidoreductase enzymes and may, under defined conditions, reflect the number of viable cells (Patel and Patel, 2011). Cells were seeded at a concentration of 1 × 10^4^ cells/well in 200 μl of Dulbecco's Modified Eagle Medium (DMEM) (Gibco) without phenol red and incubated at 37°C in 5% CO_2_. DMEM includes higher concentrations of amino acids than Eagle's Basal Medium (Gibco) so that the medium more closely approximates the protein composition of mammalian cells. After 24 h, when the monolayer formed, the medium was replaced with a final volume of 200 μl of new medium with tested compounds or controls were added to the plates. For dose-response curves, cells were treated with eight 2-fold serial dilutions of each compound spanning concentrations from 50 to 0.39 μM in 0.5% DMSO final. Controls were on the first and the last columns of the plates. On the first column, methyl methanesulfonate (MMS; Naji-Ali et al., 1994) was included as a positive control of cytotoxicity and DMSO as a negative control. On the last column there were four points of doxorubicin, another positive control of cytotoxicity (Tacar et al., 2013), with an initial concentration of 10 mM and 1:2 serial dilutions. When compounds and controls were added, plates were incubated at 37°C in 5% CO_2_ incubator for 72 h. The sample solution in wells was flicked off and 100 μl of MTT dye was added to each well. MTT solution was prepared at 5 mg/ml in PBS 1X and then diluted at 0.5 mg/ml in DMEM. The plates were gently shaken and incubated for 3 h at 37°C in 5% CO_2_ incubator. The supernatant was removed and 100 μl of DMSO 100% was added. The plates were gently shaken to solubilize the formed formazan. The absorbance was measured using a multireader VictorTM Wallac spectrofluorometer (PerkinElmer) at a wavelength of 570 nm.

## Results and discussion

### *Salmonella* biofilm assay setup

**Figure 1:**
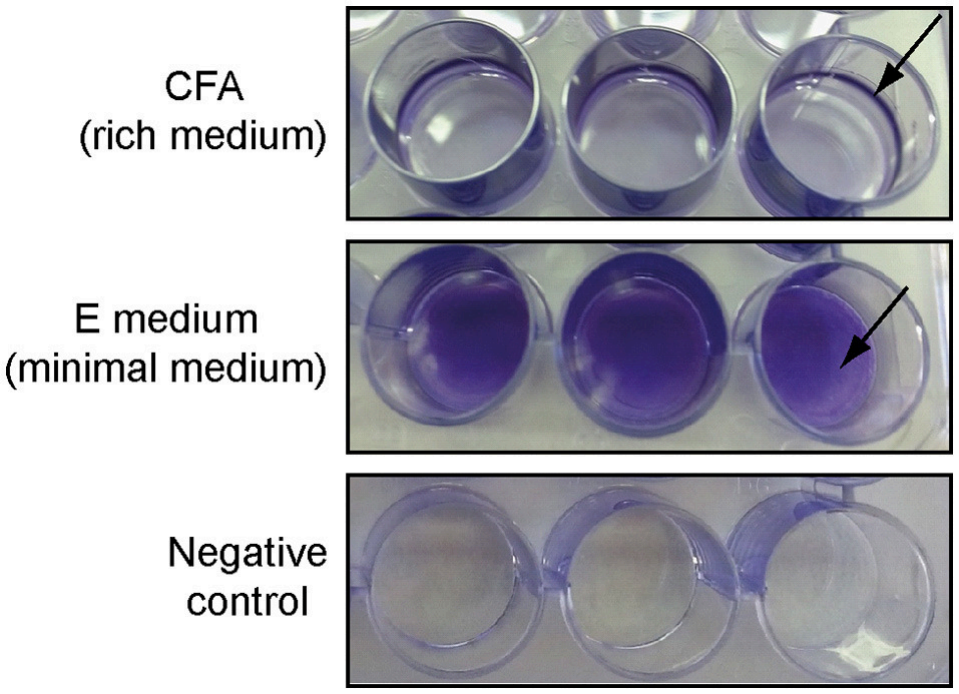
**Different types of biofilms formed by ***S***. Enteritidis 3934 when grown in 24-well-polystyrene plate in CFA (pellicle) or E medium (bottom) stained with CV after 72 h incubation at 25°C**. Negative controls are shown.

Römling and Rohde (1999) showed that *S*. Typhimurium ATCC 14028, when grown in a minimal medium, forms a so called “bottom” biofilm instead of a pellicle at the air-liquid interface. This type of biofilm is characterized by an equal distribution of cells on the solid-liquid interface (wall of the tube). Therefore, we tested whether strain 3934 was able to form bottom biofilms on polystyrene surfaces when grown in E minimal medium and standing culture. Twenty-four-well-plates were used for that purpose, and the *Salmonella* 3934 strain was inoculated in 1 ml of either CFA (rich medium) or E minimal medium. As shown in Figure 1, after 72 h incubation at 25°C, strain 3934 forms pellicle biofilms in rich media which is detected as a ring formed on the solid surface exposed to the air-liquid interphase. However, when grown in minimal medium, no pellicle biofilm was observed and a bottom biofilm was clearly detected. Bottom biofilm is easier to detect and more reliable to quantify by staining methods than pellicle biofilm. Remarkably, the observed phenomenon is not specific to *S*. Enteritidis 3934. As shown in Figure S2, the effect has also been observed on other *S. enterica* strains from serovars Typhimurium and Enteritidis. It is intriguing to note that alterations in the culture medium composition cause such relevant changes in the way a biofilm is formed by *Salmonella* (bottom or pellicle). Further studies will be required to characterize the effect of the media on biofilm formation.

### *Salmonella* biofilm assay design optimization

Once the E minimal medium was selected to be used for the biofilm formation, a standardized protocol to grow robust biofilm was defined.

The inoculum was prepared from fresh *Salmonella* 3934 grown O/N on LB-agar plates at 37°C. A cell suspension was prepared and diluted in E medium to a final OD_612_ of 0.02, which results in ~1.4 × 10^6^ cfu/well. To check the effect of a given extract on biofilm formation, 90 μl of this suspension was mixed with 10 μl of the extract for 72 h at 25°C. Since the extracts were at a concentration of 2X WBE, the resulting concentration assayed in all cases was at 1/5X WBE. Cultures were incubated at 25°C for 72 h, until a thick bottom biofilm was detected. Additionally, to analyse the activity of an extract on a pre-existing biofilm, first 100 μl of the suspension was inoculated per well and allowed to form a mature biofilm. After 72 h incubation at 25°C, wells were rinsed and 90 μl of fresh media and 10 μl of the extracts were added, as above. The incubation was extended for 24 h incubation at 25°C (see Section Materials and Methods). For untreated controls, DMSO was added to fresh media instead of extracts.

Next, antibiotic sensitivity, DMSO tolerance and the staining procedures used to monitor biofilm mass were optimized. Three technical replicas and three biological replicas were used for all validation assays.

**Figure 2:**
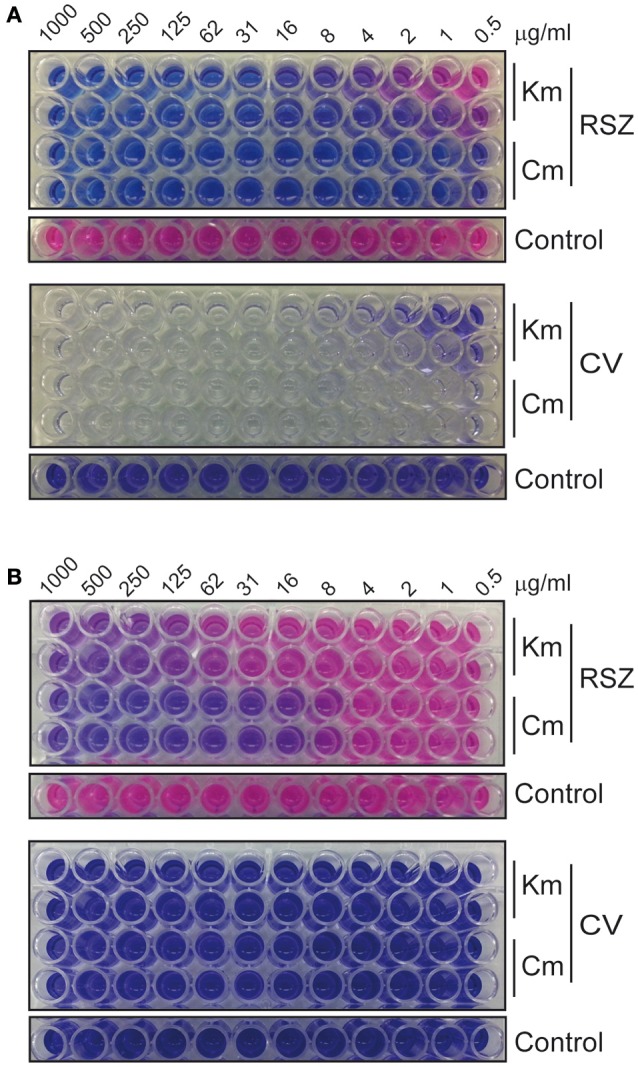
**Susceptibility dose curves with 2-fold serial dilutions of kanamycin and chloramphenicol. (A)** Biofilm formation assays allow the determination of the MIC of the antibiotics and **(B)** dispersal of preformed biofilm assays allow the determination of the MBEC. Top panels show RSZ and bottom panels show CV staining. Untreated controls are 12 replicas of the bacterial culture without the addition of antibiotic.

The optimization of the experiment, the reproducibility and the sensitivity of our method to detect deleterious effect on different stages of biofilm development, were evaluated by performing susceptibility dose curves (from 1 mg/ml to 0.5 μg/ml) with two different antibacterial compounds: kanamycin, a bactericidal antibiotic (Scholar and Pratt, 2000), and chloramphenicol, a bacteriostatic antibiotic (Scholar and Pratt, 2000). The results showed that the methodology used is appropriate to evaluate the effect of compounds that alter biofilm, both during formation of the biofilm or by dispersing preformed biofilms. The MIC was 62 μg/ml for kanamycin and 4 μg/ml for chloramphenicol (Figure 2A), whereas the MBEC for both antibiotics was above 1 mg/ml (Figure 2B). Thus, the MBEC/MIC ratios were higher than 15- and 250-fold for kanamycin and chloramphenicol, respectively. These results are in agreement with the estimated concentration of a biocide required to eliminate biofilm which are up to 100–1000 times higher compared to the concentration necessary to kill planktonic cells (Costerton et al., 1995; Stoodley et al., 2002; Høiby et al., 2010). A significant difference in antimicrobial susceptibility has also been described between the planktonic and biofilm forms of *S. enterica* strains isolated from clinical cases with gastroenteritis, with the biofilm forms showing increased resistance rates (Papavasileiou et al., 2010). The spectrum of concentrations of both antibiotics used in the assay spans from 1000 to 0.5 μg/ml. Within this range, kanamycin showed to provide a clearer increase in its inhibitory effect than chloramphenicol. For that reason, kanamycin was chosen as a control for the HTS assay.

As the HTS was going to be performed with extracts that contained a 20% DMSO, which would lead to a final concentration of 2% in the culture, the effect of DMSO on *Salmonella* 3934 biofilms was also tested. The bacteria were grown in the presence of 12 different concentrations of DMSO ranging from 10 to 0.005%. The results showed that at the working concentration of 2%, DMSO displayed low impact on biofilm formation and no effect on dispersing preformed biofilm (data not shown).

For quantitative measurement of the biofilm biomass, CV staining was performed. CV assay is cheap, straightforward and has been extensively used for the quantification of biofilms formed by a broad range of microorganisms (Peeters et al., 2008). The OD_570_ signal achieved for the untreated control stained with CV was 0.65 ± 0.07 and the ratio of the signal detected between the untreated control and the negative control was ~7, providing a wide window between controls and thus, granting a good biofilm mass quantification method for our assays. Although CV is probably one of the dyes most used for quantifying biofilm it is inadequate to assess viability of biofilm cells because matrix and both living and dead cells are stained by CV. An alternative methodology to monitor bacterial biofilm employing RSZ was also tested. RSZ is a non-toxic, water-soluble blue dye which is reduced by electron transfer reactions associated with respiration producing resorufin, a water-soluble pink product highly fluorescent (Twigg, 1945; O'Brien et al., 2000). RSZ has been used to assess viability and bacterial contamination, to test for antimicrobial activity, and as an indicator of bacterial cell numbers (Shiloh et al., 1997; Sarker et al., 2007). Over the last few years, several assays for biofilm quantification in microtiter plates have been described showing RSZ as one of the best alternatives for microbial biofilm quantification (Peeters et al., 2008; Mariscal et al., 2009). The fluorescence values achieved with RSZ for untreated controls were 9164 ± 573, showing a ratio between untreated controls and negative controls of 9, which also makes it an appropriate staining method. Quantification of biofilms by both methods, CV and RSZ, plus the determination of the OD_612_ of each well, would provide information of the specific activity of a given extract.

Since we were interested in collecting data from both staining methods, we tested whether there were significant differences between staining the biofilms with CV and staining first with RSZ and then with CV. Indeed, as shown by Skogman et al. (2012), no effect of the RSZ staining could be detected in the amount of biomass stained by CV in the same assay plate when compared to the staining performed in separate plates, as no statistical significance was detected between both staining procedures (*p* = 0.16). The OD_570_ signal achieved for untreated biofilms stained directly with CV was 0.65 ± 0.07, whereas the signal measured for the CV staining after staining with RSZ was 0.62 ± 0.13. Moreover, the ratio of the signal detected between the untreated control cells and the negative control was very similar to that observed for the CV staining, thus keeping the suitability of the assay. Therefore, to save aliquots of microbial extracts, consumables and reagents, we decided to perform the assays following the second strategy.

### Screening campaigns and hit identification

After optimization of the experimental setup, a subset of 1120 microbial extracts (560 derived from actinomycetes and 560 from fungi) from the Fundación MEDINA's collection were examined to identify molecules with antibiofilm activity by inhibiting biofilm formation, promoting dispersal of mature biofilms or both activities. Calculated parameters during the HTS resulted in Z′ factors between 0.79 and 0.97 and RZ′ factors comprised between 0.86 and 0.98, indicating the high robustness and reproducibility of the HTS assay. From the primary screening, different degrees of inhibition were detected.

Using the data obtained with RSZ quantification, extracts were selected when they displayed at least a 90% inhibition on the biofilm formation assay. Using this criterion 32 extracts were selected, 11 from actinomycetes, and 20 from fungi. On the other hand, when looking at the effect of the extracts on the biofilm dispersal assay, lower levels of metabolic activity inhibition were detected. In this case, a threshold of 45 and 35% inhibition was used for actinomycetes and fungal extracts, respectively. Consequently, 14 extracts from actinomycetes and eight from fungi were selected. Remarkably, three of the above mentioned extracts showed effects on both formation and dispersal of the biofilm (Table 1). Altogether, 50 extracts from a total of 1120 assayed (4.46%) were initially identified as anti-*Salmonella* biofilm hits for further studies.

**Table 1:** **Number of extracts identified as hits at different stages of the screening process**.

	**Primary HTS**	**Cherry-picking**	**<30% HepG2 Cell Death**	**Prioritized for refermentation and activity confirmation**
Actinomycetes	24	20	11	3
F	10	8	5	2 (#33 and #35)
D	13	11	5	
F+D	1	1	1	1 (#31)
Fungi	26	20	8	2
F	18	15	6	
D	6	3	2	1 (#7)
F+D	2	2	–	1 (#25)
Total	50	40	19	5

*F, Formation; D, Dispersal; F+D, Formation and Dispersal*.

A cherry-picking campaign was carried out to confirm the activity and reproducibility of the effect on biofilms of the natural extracts and confirmed 40 of the initial hits (Table 1 and Table S1).

Additionally, cytotoxicity of the extracts was tested using the hepatic cell line HepG2. A total of 19 extracts (Table S1) was found to inhibit <30% growth of HepG2 cells when tested at 1/40X WBE final concentration for 72 h.

### Small-scale regrowths

As described by others (Sandberg et al., 2008, 2009; Skogman et al., 2012; Fallarero et al., 2013; Manner et al., 2013), by combining the data on biofilm quantification by both RSZ and CV staining methods and the value of OD_612_ of the culture, more specific information on the activity of the extracts can be obtained. As shown in Table 2, we define four types of activities amongst the extracts, which were not mutually exclusive, that is: (1) inhibition of biofilm formation, (2) antimicrobial activity of cells in suspension, (3) antimicrobial activity of cells that are part of the biofilm, and (4) dispersal of the biofilm.

**Table 2:** **Antibiofilm activities detected at the HTS**.

		**OD_612_**	**Resazurin**	**Crystal violet**
Untreated		+++	+++	+++
Formation	1	+++	+	+
	2	+	+	+
Dispersal	3	n.d.	+	+++
	4	n.d.	+	+

*n.d., not determined*.

*+ and + + + refers to low and high levels of the specified parameter, respectively*.

At this stage, three extracts from bacterial cultures (#31, #33, and #35) and one extract from fungal cultures (#7)—identified as containing potentially novel active components—were prioritized. Two of them were able to inhibit biofilm formation (#33 and #35); one extract had antimicrobial activity against cells in suspension and cells within a biofilm (#31); and extract #7 was able to disperse biofilm. Additionally, we also prioritized extract #25, which in spite of showing a clear cytotoxicity also retained a very strong antimicrobial effect on both planktonic and sessile cells (Table 3). It is worth mentioning that natural extracts may contain hundreds of different compounds. Thus, the cytotoxic activity of extract #25 on HepG2 cells might be caused by compounds different to those showing an antibiofilm effect. The percentage of inhibition of the biofilm at the different points of the screening for the four extracts prioritized plus extract #25 is shown in Table 3, as well as two extracts with no antibiofilm activity for reference.

**Table 3:** **Activities shown by the extracts prioritized for refermentation and activity confirmation**.

	**BIOFILM FORMATION ASSAY Screening**				**BIOFILM FORMATION ASSAY Cherry–picking**				**BIOFILM DISPERSAL ASSAY Screening**				**BIOFILM DISPERSAL ASSAY Cherry–picking**				**ACTIVITY CODE*^a^***				**CYTOTOXICITY**
	**DAY 1**		**DAY 2**		**DAY 1**		**DAY 2**		**DAY 1**		**DAY 2**		**DAY 1**		**DAY 2**		**1**	**2**	**3**	**4**	
**#**	**% viability**	**% total biomass**	**% viability**	**% total biomass**	**% viability**	**% total biomass**	**% viability**	**% total biomass**	**% viability**	**% biofilm biomass**	**% viability**	**% biofilm biomass**	**% viability**	**% biofilm biomass**	**% viability**	**% biofilm biomass**					**HepG2 % Cell death**
7 (D)	–8	–43	4	28	–17	71	–17	16	–46	–48	–35	–48	–21	–45	–31	–44				•	–26
25 (F+D)	–100	–103	–102	–99	–101	–110	–100	–101	–92	–35	–93	–43	–70	–29	–64	–28		•	•		–100
31 (F+D)	–93	–96	–92	–101	–70	63	–58	76	–52	–56	–47	–53	–36	–50	–40	–49		•	•		17
33 (F)	–98	213	–99	331	–97	141	–99	228	–24	1	–11	18	–5	66	15	19	•				–6
35 (F)	–100	145	–100	159	–100	269	–100	340	–12	9	–5	23	–4	23	20	14	•				6
FNG C–	20	87	24	88	n.d.	n.d.	n.d.	n.d.	0	–1	–1	4	n.d.	n.d.	n.d.	n.d.					
ATM C–	–6	181	1	238	n.d.	n.d.	n.d.	n.d.	–12	–15	11	–8	n.d.	n.d.	n.d.	n.d.					

a *(1) Inhibition of biofilm formation, (2) Antimicrobial activity of cells in suspension, (3) antimicrobial activity of cells that are part of the biofilm and (4) dispersal of the biofilm*.

*n.d. not determined. The scale of colors indicate the percentage of viability, from red (less viability) to green (more viability)*.

*F, Formation; D, Dispersal; F+D: Formation and Dispersal*.

*Last two rows show the activity of two extracts from actinomycete (ATM C–) and fungi (FNG C–) with no activity. The data indicates the percentage of inhibition of biofilm formation determined by resazurin (viability) and OD_612_ measures (total biomass), and the percentage of dispersal of preformed biofilm determined by RSZ (viability) and CV staining (biofilm biomass), in the screening and cherry–picking campaigns. Day 1 and day 2 refers to the data obtained independently in two different days. The type of activity of each extract is indicated with a black circle (•) according to the code assigned in the text*.

A database search, which matches UV-LC-MS data of the metabolites in the active extracts to UV-LC-MS data of known metabolites, did not show any known or conclusive metabolite, suggesting that they were likely to contain novel potentially active components. Thus, the same producing bacterial and fungal cultures were regrown in 10 ml and 100 or 150 ml volumes (for fungal and bacterial cultures, respectively) in the same production conditions and the antibiofilm activity of the newly generated crude extracts was assessed. The activity of the five natural extracts was confirmed (data not shown).

### Dose-response experiments

**Figure 3:**
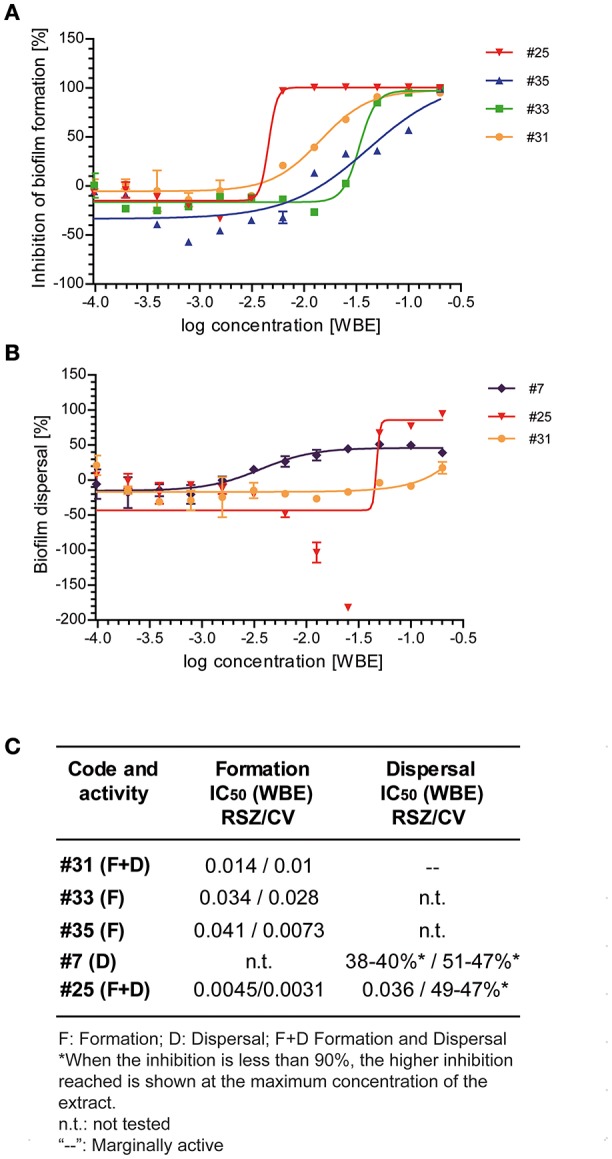
**Dose-response curves of the selected extracts identified for its activity inhibiting biofilm formation (#25, #35, #33, and #31) or its activity dispersing preformed biofilms (#7, #25, and #31)**. Dose-response curves of the indicated extracts on biofilm formation assays **(A)** or on biofilm dispersal assays **(B)**. The inhibitory activity of each extract was determined by RSZ staining. Mean and standard error of mean of two independent experiments is shown. **(C)** IC_50_-values obtained after staining with RSZ and CV are shown in whole broth equivalents of the selected natural extracts.

To assess the potency of the five extracts selected, we performed dose-response experiments with the small-scale regrowth extracts. The extracts were tested in a 12-point dose-response assay, being the first point the initial dilution previously described for each assay (1/5X WBE, final concentration) followed by the 11 subsequent 2-fold serial dilutions (Figure 3).

Dose-response assays performed confirmed the activity of the extracts tested inhibiting biofilm formation (Figures 3A,C). The most potent extract was #25, which showed an IC_50_ between 3 and 10 times lower than the rest of the extracts previously proved to retain this activity (#31, #33, and #35). This extract maintains an inhibition percentage of 90% up to the 64-fold dilution of the original extract, whereas the other extracts dropped their activity after the 2-fold (#35) or the 8-fold (#31 and #33) dilution of the initial extract.

When extracts #7, #25, and #31 were tested for their activity dispersing preformed biofilm (Figures 3B,C), we could observe that much higher concentrations were required to detect antibiofilm activity. Moreover, we were only able to calculate the IC_50_ for extract #25, since the other two extracts did not reach a 100% dispersal of the preformed biofilm. Extract #25 showed an increase of the IC_50_ of ~8-fold, compared to its inhibitory activity on biofilm formation. However, our data suggest that extract #25 contains a very potent compound that can efficiently affect biofilm cells compared to other antibiotics. As shown in this report for kanamycin and chloramphenicol and in other studies (Costerton et al., 1995; Stoodley et al., 2002; Høiby et al., 2010), antibiotics are largely inefficient against bacteria grown in biofilms. Notoriously, the IC_50_ for this extract was one dilution higher when stained with CV than with RSZ, indicating a reduction of viability of the cells embedded in the biofilm rather than a dispersal of the total biofilm biomass. Although extract #7 was chosen for its specific activity against preformed biofilms, in contrast to extract #25, at the maximum concentration it was only able to reduce a 40% of the living cells within the biofilm. Similarly to extract #7, it was not possible to calculate the IC_50_ of extract #31, since the maximum activity reached was much lower than 100%.

Interestingly, the dose-response curve displayed by extract #25 on biofilm dispersal shows an increase in the metabolic activity at concentrations immediately below the IC_50_. The U-shaped dose-response observed is known as hormesis, a phenomenon characterized by a low dose stimulation and high dose inhibition, and it has been extensively described for antibiotics (Davies et al., 2006). The behavior observed by extract #25 was detected by RSZ staining method but not by CV staining (data not shown), suggesting that, at sublethal concentrations, active compounds contained in this extract cause a bacterial stress that is associated to the high metabolic activity rather than an increase of the total biomass of the biofilm.

### Fractionation of the small-scale regrowths, de-replication of active fractions, and dose-response experiments

To identify the antimicrobial compound present in extract #25, extracts from 10 to 100 ml cultures were fractionated as indicated in Materials and Methods Section. Two fractions were shown to retain the same activity as the original natural extract. A database search performed using UV-LC-MS data, identified patulin in the two active fractions of extract #25. We were not able to identify patulin in whole extract #25, indicating the high complexity of this extract. Patulin is a fungal mycotoxin produced by certain molds of the genera *Penicillium, Aspergillus* and *Byssochlamys* growing on a variety of foods including fruit, grains, and cheese (Sommer et al., 1974; U.S. Food and Drug Administration). Patulin, also known as clavacin, claviformin, expansin, mycoin C, and penicidin, shows antibiotic activity against Gram-negative and Gram-positive bacteria (reviewed in Hopkins, 1943; Florey et al., 1949). Although it has been reported that patulin shows cytotoxic activity against some cell lines (Puel et al., 2010; Song et al., 2014; Figure S3), according to the International Agency for Research on Cancer (IARC, http://www.iarc.fr), patulin is classified in the group 3 as “not classifiable as to its carcinogenicity to humans” (International Agency of Research on Cancer, 1986).

Based on the commercial availability of patulin, we decided to test whether this compound was the responsible of the inhibitory activity on *Salmonella* biofilms performing dose-response assays. As a control, we used kanamycin, a broad-spectrum antibiotic against many Gram-negative and Gram-positive bacteria (Takeuchi et al., 1957), and ciprofloxacin, a broad-spectrum second generation fluoroquinolone (King et al., 2000) and the most often used antibiotic against *Salmonella* non-typhoidal infections (Gilbert et al., 2016).

**Figure 4:**
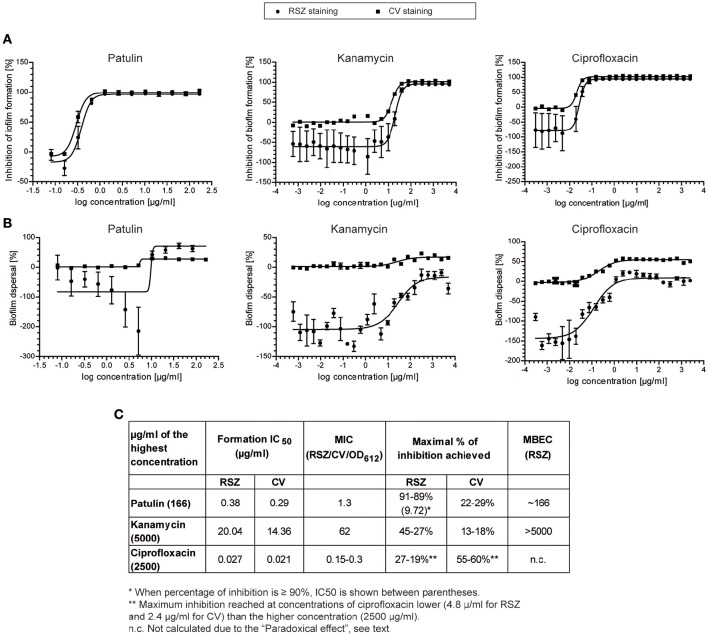
**Dose-response curves of patulin, kanamycin, and ciprofloxacin**. The inhibitory activity in biofilm formation assay **(A)** or in biofilm dispersal assay **(B)** was determined by RSZ (•) or CV (■) staining. Mean and standard error of mean of two independent experiments is shown. **(C)** IC_50_, MIC, and MBEC-values (μg/ml) for patulin and the two broad-spectrum antibiotics used are shown.

As shown in Figures 4A,C, patulin, kanamycin, and ciprofloxacin prevented biofilm formation with a MIC of 1.3, 62, and 0.3 μg/ml, respectively, as monitored by both RSZ (living cells) and CV (biomass) determination. At concentrations of the compounds higher than the MIC, we also observed a decrease of the OD_612_, clearly indicating that all the tested compounds kill the planktonic cells, thus avoiding biofilm formation. This indicates an antimicrobial behavior of the compounds tested rather than an inhibition of biofilm formation. According to that observation and in spite of the small differences between the data obtained from both staining methods, similar results from both assays were seen. The calculated IC_50_ and MIC indicate that ciprofloxacin is the most potent antibiotic of the assay, with an activity between 5- and 14-fold higher than patulin, respectively (Figure 4). Patulin shows higher antimicrobial activity than kanamycin since it showed a MIC of 1.3 μg/ml, which is in the order of 50-fold more potent than kanamycin. Also, the ratios between IC_50_ of both compounds were similar to the ratios shown for the MIC. It is noteworthy mentioning that, isolated compounds with MICs <10 μg/ml are considered very interesting as antimicrobial agents (Ríos and Recio, 2005).

When looking at the activities of the compounds tested on preformed biofilms, we observed that patulin treatment produce a 90% decrease of RSZ staining (cell viability) but a 30% decrease of CV staining (cell biomass). This effect can be explained by the fact that RSZ only monitors living bacteria and the dead cells that are stained with CV are not taken into account (Skogman et al., 2012). Thus, patulin activity on preformed biofilms is likely to be through killing bacteria embedded into the biofilm which leads to a partial dispersion of the biofilm.

Moreover, when assaying the dose-response curve of patulin on biofilm dispersal assays (Figure 4B) we detected a phenomenon similar to that observed in extract #25. Patulin causes an eustress- a beneficial stress followed by an inhibitory activity—at sublethal concentrations, that is at concentrations between 5.2 and 1.3 μg/ml. Therefore, we may conclude that the hormesis event observed in extract #25 was likely to be carried out by patulin. It is noteworthy mentioning that patulin was found to enhance biofilm formation in *P. aeruginosa* in a concentration dependent manner up to the maximal concentration of 25 μM (Liaqat et al., 2010) which corresponds to 3.8 μg/ml. They also observed that patulin prolonged the lag phase in *P. aeruginosa* at concentrations >10 μM (1.54 μg/ml). More recently, the effect of patulin was tested on *Halomonas pacifica* and *Marinobacter hydrocarbonoclasticus*, two halophilic marine micro-organism involved in biofouling processes (Liaqat et al., 2014). Biofilm formation on *H. pacifica* was enhanced with increasing patulin concentration up to 10 μM and then decreased significantly with maximum inhibition at 25 μM concentration. This behavior corresponds to the eustress caused by patulin that we also observe. No significant effect of patulin was observed on *M. hydrocarbonoclasticus*. We would like to highlight that the concentrations used in both works are in the range of concentrations observed in this study that cause the eustress effect on *Salmonella*. The fact that patulin shows an enhancing effect on *P. aeruginosa* biofilms or no effect on *M. hydrocarbonoclasticus* biofilms might be due to the concentrations used in the assay, which are probably in the eustress zone or below the sublethal concentration, respectively. Interestingly, patulin has been described as a biologically active quorum-sensing inhibitory (QSI) compound in *P. aeruginosa* (Rasmussen et al., 2005). The target of patulin in *Salmonella* remains unknown and deserves further research.

Remarkably, the comparison of the metabolic activities (RSZ staining) of the *Salmonella* preformed biofilms treated with the tested compounds, exhibited patulin as the most potent antimicrobial assayed. Patulin is more than 15-fold stronger than ciprofloxacin on killing biofilm cells, since 166 μg/ml were needed to obtain a 90% reduction, whereas 27–19% was the maximum inhibitory activity reached for ciprofloxacin at a concentration of 4.8 μg/ml. At higher concentrations than 4.8 μg/ml, the inhibitory activity of ciprofloxacin shows a slight decrease. The reduction in bactericidal activity with concentrations of antibiotics above a critical level has been described as “paradoxical effect” and it is well-documented for ciprofloxacin and other quinolones (Dörr et al., 2009; Malik et al., 2009). Moreover, in the case of ciprofloxacin, the reduction of preformed biofilm detected was higher when the biofilm was stained with CV. In this case, the maximal activity detected with CV after treatment with ciprofloxacin starts at 2.4 μg/ml and from this point, the inhibitory activity hit a plateau.

In summary, the HTS designed reveals to be a powerful tool for the identification of novel NPs with antimicrobial activity on *Salmonella* biofilms. While similar platforms have already been described to identify different types of antimicrobial activity of a given compound (Sandberg et al., 2008, 2009; Skogman et al., 2012; Fallarero et al., 2013; Manner et al., 2013), this approach identifies compounds that inhibit biofilms of the relevant pathogen *Salmonella*. Moreover, it must be pointed up that the developed HTS uses a collection of NPs to search for new antibiofilm compounds. The pilot screening used to validate the methodology, drove us to the identification of patulin as a molecule that efficiently kills *Salmonella* cells within a biofilm. Indeed, the biocide effect of patulin on biofilm embedded bacteria is much more powerful than the observed for ciprofloxacin, the most common antibiotic used for treatment of salmonellosis. Patulin cytotoxicity against some cell lines has been reported (Puel et al., 2010; Song et al., 2014) and this might limit its use in clinical practice. Although further studies will be required to determine its possible use for biomedical purposes, patulin has a great potential as disinfectant. We would like to highlight that HTS has been performed at 25°C, temperature that mimics environmental conditions rather than in-host environment. In this report, we describe and validate an HTS assay to identify new and effective antibiofilm molecules. This approach can potentially provide, as exemplified by the finding of patulin during the pilot screening, tools to reduce costs of disinfection of industrial facilities and to improve the food quality, and safety of the final products.

## Conclusions

We have identified and validated the growth conditions for *S*. Enteritidis to grow forming a bottom biofilm. These findings were central to establish a robust, reliable, and comprehensive HTS platform for the identification of potentially novel NP-based drug leads which either can inhibit the formation or can eliminate or disperse preformed biofilms of *Salmonella*. This platform has been used in a pilot project to screen 1120 microbial extracts and has confirmed 40 extracts as having potential novel antibiofilm metabolites. Four of them are currently under study for the identification of novel hit components and we hope they can deliver new molecules active against *Salmonella* biofilms. The pilot screening performed opens the possibility to perform screenings with a much larger collection of NPs. This approach meets the demanding requirements of HTS drug discovery, and also it has been validated with the identification of patulin as component of one of the bioactive extracts.

## Author contributions

SP, OG, FR, FV, CM, and CB conceived the research. SP and Md responsibles of setting up the assays, data analysis, and interpretation of the results. IG and VG carried out the fermentation and the taxonomic identification of the microbial strains. JT performed the extraction of the culture broths and fractionation of the extracts. JM performed the LC/MS analyses and analyzed and interpreted the ESI-TOF spectra. Md, OG, FR, and FV critical revision of the article. SP, CM, and CB wrote the manuscript.

## Funding

This work was supported by grants from the RecerCaixa program (2012/ACUP/00048), the Spanish Ministry of Science and Innovation (AGL2013-45339-R), and the Generalitat de Catalunya (2014SGR1260).

### Conflict of interest statement

The authors declare that the research was conducted in the absence of any commercial or financial relationships that could be construed as a potential conflict of interest.

## Supplementary material

Figure S1 **Set of control wells used in the HTS assays, located in the left (negative and untreated controls), and right (kanamycin dose-response curve) columns of the 96-well-plates**.

Figure S2 **Different types of biofilms formed by ***S***. Enteritidis 3934 and 164372.7 and ***S***. Typhimurium HC142156206, SV5015, and ATCC 14028**. Cells were grown in 24-well-polystyrene plate in E medium (bottom biofilm) or CFA (pellicle) and stained with CV after 72 h incubation at 25°C. Negative controls for each media are shown.

Figure S3 **Dose-response curve of the cytotoxic activity of patulin on hepatic cell line HepG2 cells after 72 h treatment**. The IC_50_-value obtained was 0.46 μM.

Table S1 **Activities shown by the 40 pre-selected extracts confirmed by cherrypicking**. The data indicates the percentage of inhibition of biofilm formation determined by resazurin (viability) and OD612 measures (total biomass), and the percentage of dispersal of preformed biofilm determined by resazurin (viability) and CV staining (biofilm biomass), in the screening and cherry-picking campaigns. Day 1 and 2 refers to the data obtained independently in 2 different days. In red, cytotoxicity levels on HepG2 cells higher than 30% are shown.
