# Supplementary figures and images for: A High-Throughput Screening Platform of Microbial Natural Products for the Discovery of Molecules with Antibiofilm Properties against Salmonella
Source: Front Microbiol. 2017 Mar 2;8:326. doi: 10.3389/fmicb.2017.00326 (PMC5332434; doi:10.3389/fmicb.2017.00326)

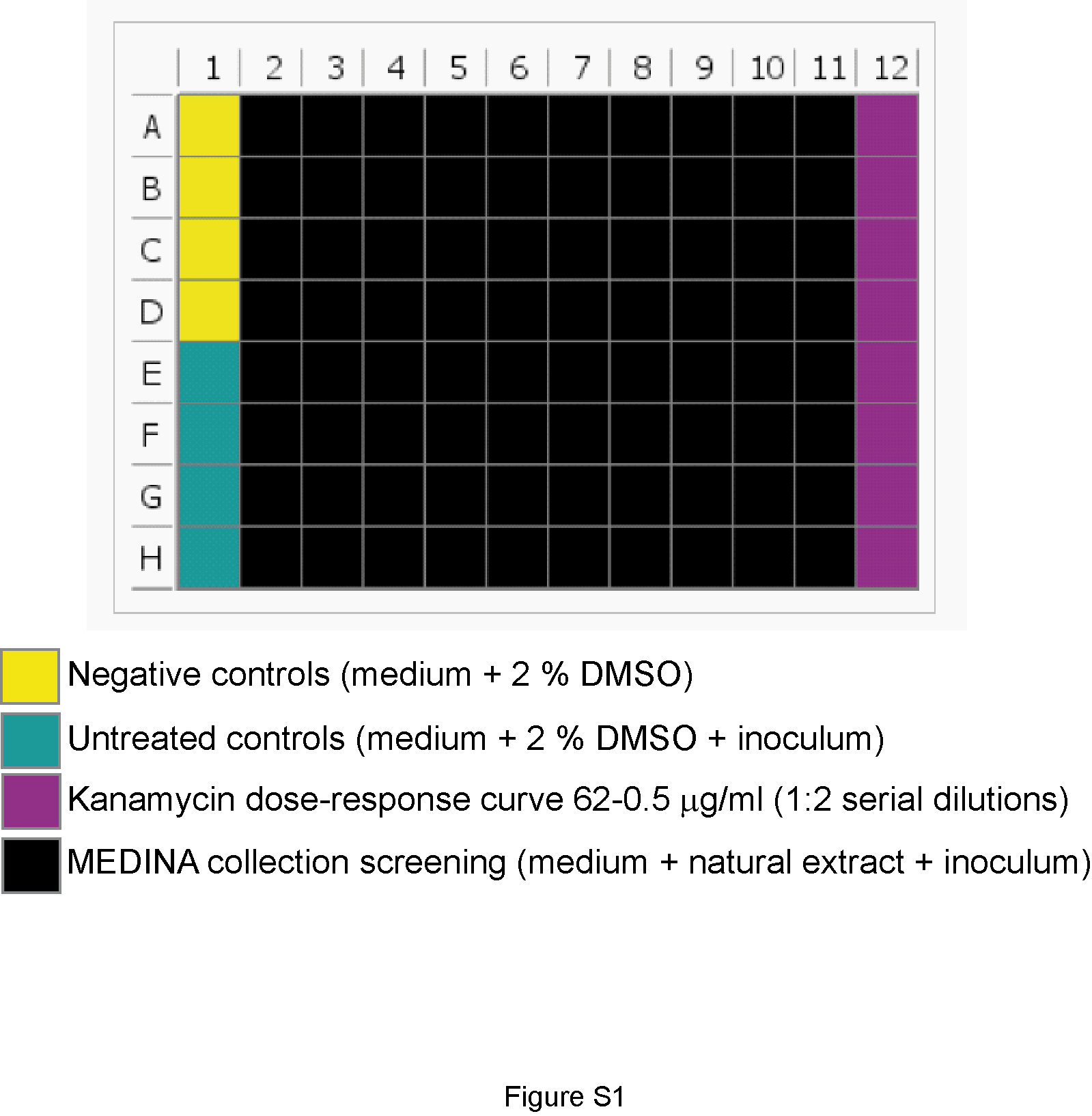

Supplement: Figure S1 — Set of control wells used in the HTS assays, located in the left (negative and untreated controls), and right (kanamycin dose-response curve) columns of the 96-well-plates. [file Image1.TIF]

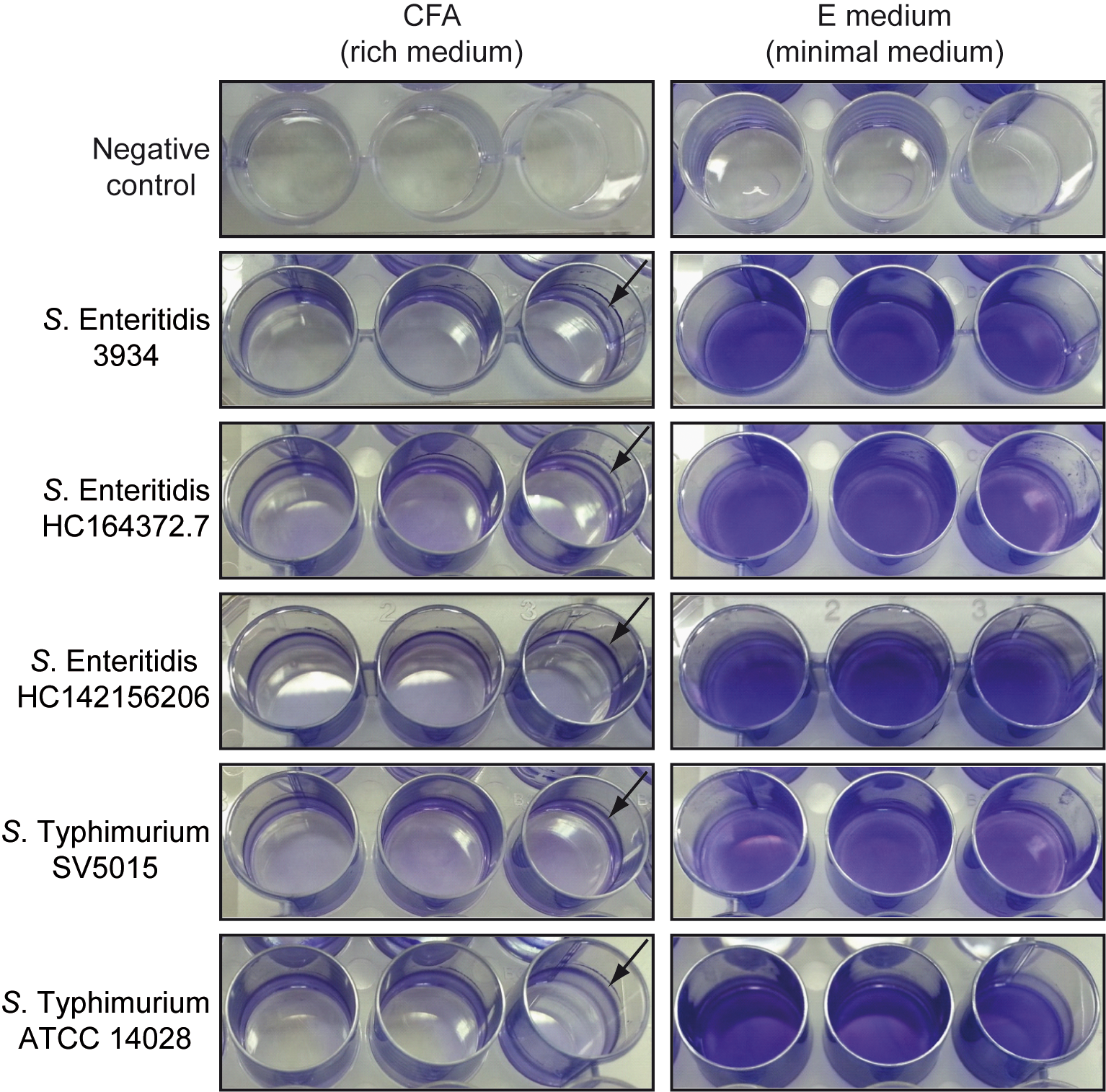

Supplement: Figure S2 — Different types of biofilms formed by S. Enteritidis 3934 and 164372.7 and S. Typhimurium HC142156206, SV5015, and ATCC 14028. Cells were grown in 24-well-polystyrene plate in E medium (bottom biofilm) or CFA (pellicle) and stained with CV after 72 h incubation at 25°C. Negative controls for each media are shown. [file Image2.TIF]

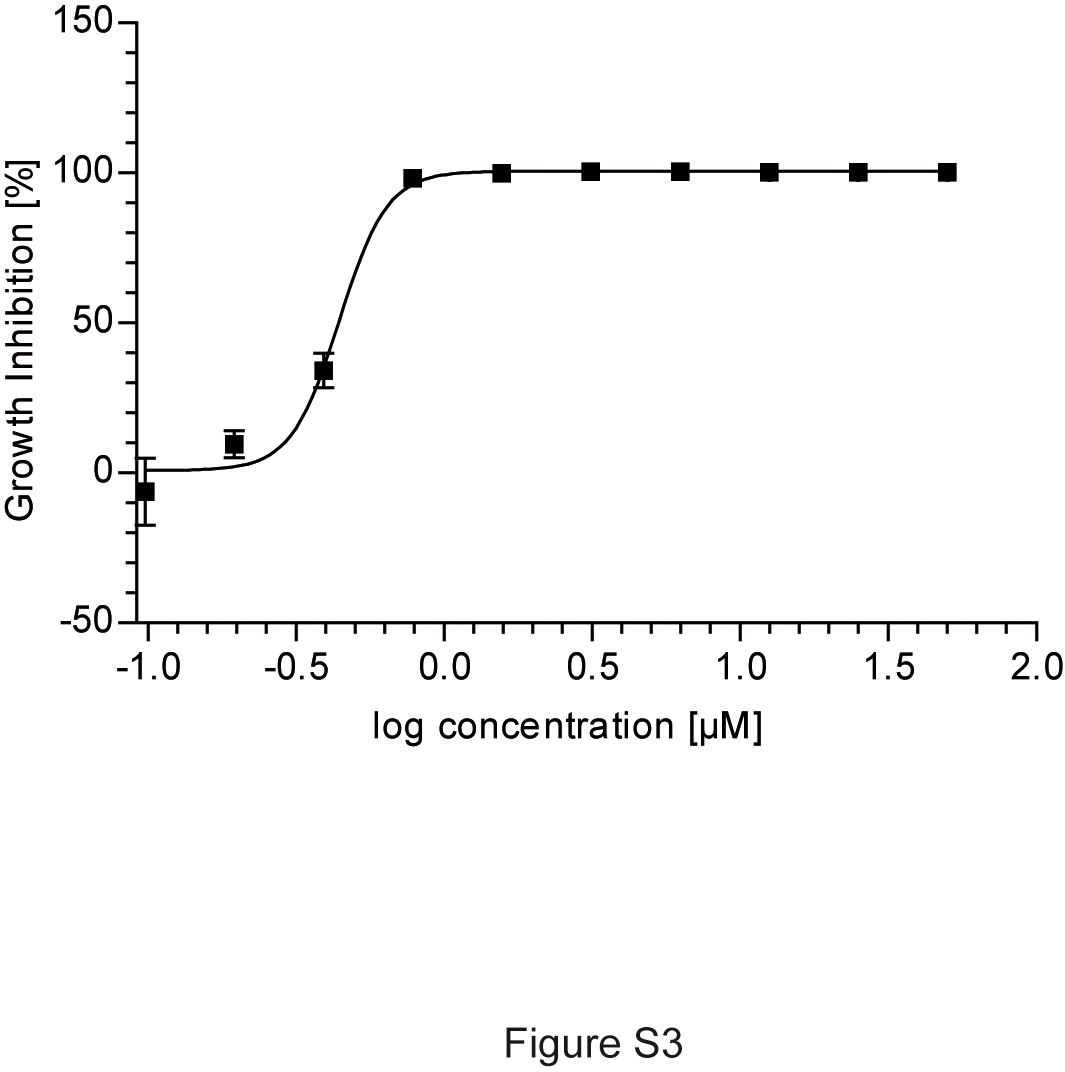

Supplement: Figure S3 — Dose-response curve of the cytotoxic activity of patulin on hepatic cell line HepG2 cells after 72 h treatment. The IC50-value obtained was 0.46 μM. [file Image3.TIF]
